# Aβ-Targeting Bifunctional Chelators (BFCs) for Potential Therapeutic and PET Imaging Applications

**DOI:** 10.3390/ijms24010236

**Published:** 2022-12-23

**Authors:** Olga Krasnovskaya, Aina Kononova, Alexander Erofeev, Peter Gorelkin, Alexander Majouga, Elena Beloglazkina

**Affiliations:** 1 Department of Materials Science of Semiconductors and Dielectrics, National University of Science and Technology (MISIS), Leninskiy Prospect 4, 119049 Moscow, Russia; 2 Chemistry Department, Lomonosov Moscow State University, Leninskie Gory 1-3, 119991 Moscow, Russia

**Keywords:** Alzheimer’s, PET, SPECT, amyloid

## Abstract

Currently, more than 55 million people live with dementia worldwide, and there are nearly 10 million new cases every year. Alzheimer’s disease (AD) is the most common neurodegenerative disease resulting in personality changes, cognitive impairment, memory loss, and physical disability. Diagnosis of AD is often missed or delayed in clinical practice due to the fact that cognitive deterioration occurs already in the later stages of the disease. Thus, methods to improve early detection would provide opportunities for early treatment of disease. All FDA-approved PET imaging agents for Aβ plaques use short-lived radioisotopes such as ^11^C (t_1/2_ = 20.4 min) and ^18^F (t_1/2_ = 109.8 min), which limit their widespread use. Thus, a novel metal-based imaging agent for visualization of Aβ plaques is of interest, due to the simplicity of its synthesis and the longer lifetimes of its constituent isotopes. We have previously summarized a metal-containing drug for positron emission tomography (PET), magnetic resonance imaging (MRI), and single-photon emission computed tomography (SPECT) imaging of Alzheimer’s disease. In this review, we have summarized a recent advance in design of Aβ-targeting bifunctional chelators for potential therapeutic and PET imaging applications, reported after our previous review.

## 1. Introduction

Alzheimer’s disease (AD) is a multifactorial neurodegenerative disorder, which is characterized by a number of hallmarks, such as cerebral deposition of amyloid β-protein (Aβ) and intracellular neurofibrillary tangles (NFTs) formed by tau protein, neuroinflammation and loss of cholinergic neurons [1,2]. Aβ is produced from amyloid precursor protein (APP), which is formed from cleavages by β-secretase and γ-secretase, which leads to the formation of two predominant Aβ alloforms, Aβ_40_ and Aβ_42_ [3]. Thus, Aβ_42_/Aβ_40_ blood level is widely used as a biomarker of PET status of AD patients [4]. In addition, soluble Aβ oligomers have been shown to be involved in the synapse loss and neuronal injury [5]. The formation of Aβ-metal conjugates is often accompanied by the generation of reactive oxygen species (ROS) through Fenton chemistry, which in turn leads to enhanced oxidative stress [6].

Among the various imaging modalities such as magnetic resonance imaging (MRI) and computerized tomography (CT), positron emission tomography (PET) and single photon emission computed tomography (SPECT) are extensively used in the diagnosis of neurological disorders [7]. MRI and PET are the most frequently used imaging techniques in clinical settings. However, MRI has low detection sensitivity and can only visualize the larger plaques or tangles (>50 μm) with long acquisition time [8]. Compared with MRI, radiolabeled PET and SPECT probes have high sensitivity and can visualize most interactions between physiological targets and ligands [9]. In addition, optical imaging of Aβ plaques is of high interest due to several undeniable advantages, such as being non-invasive, non-radioactive, and inexpensive [10,11]. However, optical imaging is still limited by weak penetration, especially considering the fact that Aβ plaques and tau proteins are buried inside the brain [12].

**Figure 1:**
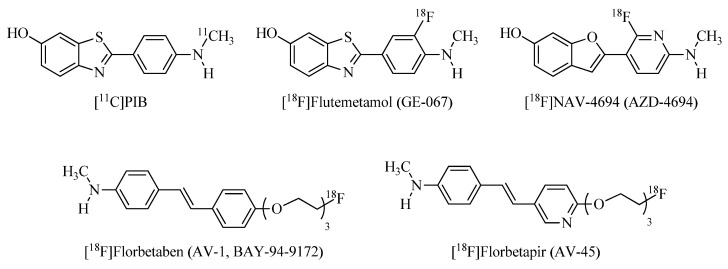
FDA-approved drugs for PET-imaging of amyloid plaques: Pittsburgh Compound-B ([^11^C]PIB), [^18^F]flutemetamol ([^18^F]GE-067), [^18^F]NAV-4694 (AZD-4694), [^18^F]florbetaben ([^18^F]AV-1, [^18^F]BAY-94-9172), and [^18^F]florbetapir ([^18^F]AV-45).

The first PET in vivo imaging of Aβ in an AD patient was performed in 2002 with the ^11^C-labeled Pittsburgh compound B ([^11^C]PIB, Figure 1), a radiolabeled PET traced based on Aβ staining agent thioflavin-T (ThT) [13]. To date, [^11^C]PIB is still a gold standard for non-invasive amyloid imaging in humans. However, the short half-life of the ^11^C isotope (T_1/2_ = 20 min, β+ ≈ 100%, Emax = 0.96 MeV) was a stimulus for the design of a novel PET-tracers labeled with longer-lived nuclides. Widely used in clinical practice, the ^18^F isotope possesses the longer half-life (T_1/2_ = 110 min, β^+^ = 97%, Emax = 0.63 MeV), which greatly simplifies both the synthesis of radiopharmaceuticals based on it and its clinical use.

Therefore, there are several ^18^F-based radioligands with favorable binding and imaging properties, [^18^F]florbetapir ([^18^F]AV-45), [^18^F]florbetaben ([^18^F]AV-1, [^18^F]BAY-94-9172), and [^18^F]flutemetamol ([^18^F]GE-067) that have also been approved by the United States Food and Drug Administration (FDA) for clinical diagnosis of AD [14,15,16,17,18,19] (Figure 1).

However, these PET imaging agents are still labeled with short-lived radioisotopes, and a production of these isotopes makes PET diagnostics dependent on cyclotron location and limits the use of radiopharmaceuticals [20,21]. In addition, radiolabeling schemes of ^11^C and ^18^F complexes often require complex multistep synthesis.

Among the Aβ imaging products being developed, special attention is paid to coordinating copper compounds for PET imaging of amyloid plaques. Copper cations seem to be one of the main cationic elements in Aβ plaque formation, and Cu^2+^ has been shown to stabilize soluble neurotoxic Aβ species [22]. One copper radionuclide, ^64^Cu (t_1/2_ = 12.7 h, β^+^ = 17%, β^–^ = 39%, electron capture EC = 43%, and Emax = 0.656 MeV) has a unique decay profile and can be used for positron emission tomography imaging and radionuclide therapy. The well-established coordination chemistry of copper allows for its reaction with different types of chelator systems [23]. Thus, several ^64^Cu-based coordination compounds were successfully used in vivo for the PET imaging and diagnosis of tumors [24] and hypoxia [25].

In addition, ^68^Ga (T_1/2_ = 68 min, β^+^ = 89%, E_max_ = 1.92 MeV) is a generator produced positron-emitting radionuclide, thus allowing for the distribution of PET imaging agents independent of on-site cyclotrons [26]. Further., the complex formation reaction is simple, does not require the synthesis of radiolabeled ligands, and allows convenient introduction of a radioactive label at the last stage of the synthesis, which favorably distinguishes metal-containing radiopharmaceuticals from those based on ^11^C and ^18^F.

We have previously summarized a metal-containing drug for positron emission tomography (PET), magnetic resonance imaging (MRI), and single-photon emission computed tomography (SPECT) imaging of Alzheimer’s disease [27]. In this review, we summarize a recent advance in design of Aβ-targeting bifunctional chelators for potential therapeutic and PET imaging applications.

## 2. Bifunctional Chelators for Visualization of Aβ Plaques

Aβ aggregates possess amphiphilic properties, including hydrophobic cores and water-soluble hydrophilic regions [28]. Thus, a conjugation of hydrophilic moieties to hydrophobic Aβ fibril-binding fragments is an effective strategy to design Aβ-targeted ligands, as such an amphiphilic molecule can interact with both the hydrophobic regions and the hydrophilic residues of the soluble Aβ oligomers. In addition, as AD is a complex disorder with multiple pathogenic factors, a novel paradigm for AD treatment is the design of multifunctional compounds (MFCs). Thus, both for PET imaging agent design and for anti-AD drugs, a common approach is a development of bifunctional chelators (BFCs) via bioconjugation of a metal chelator that forms highly stable complexes with Aβ-targeting aromatic moiety [29,30,31].

For Aβ-affinic aromatic moiety, a number of fibril-specific dyes are commonly used, such as Congo Red (CR) or ThT. Despite the fact that neither CR nor ThT are suitable for in vivo application, they serve as the promising scaffolds for development of improved imaging agents to detect amyloid accumulation [32].

**Figure 2:**
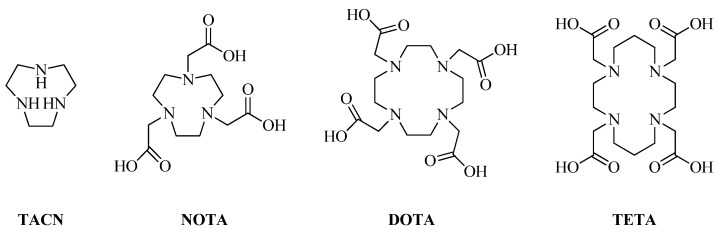
Commonly used cyclic copper chelators TACN, NOTA, DOTA, and TETA.

For copper chelators, cyclic chelators such as 2,4,7-triazacyclononane (TACN), 1,4,7-triazacyclononane-1,4,7-triacetic acid (NOTA), 1,4,8,11-tetraazacyclotetradecane-N,N′,N′′,N′′′-tetraacetic acid (TETA), and 2,2′,2′′,2′′′-(1,4,7,10-tetraazacyclododecane-1,4,7,10-tetrayl)tetraacetic acid (DOTA) are usually used [33,34,35,36] (Figure 2).

**Figure 3:**
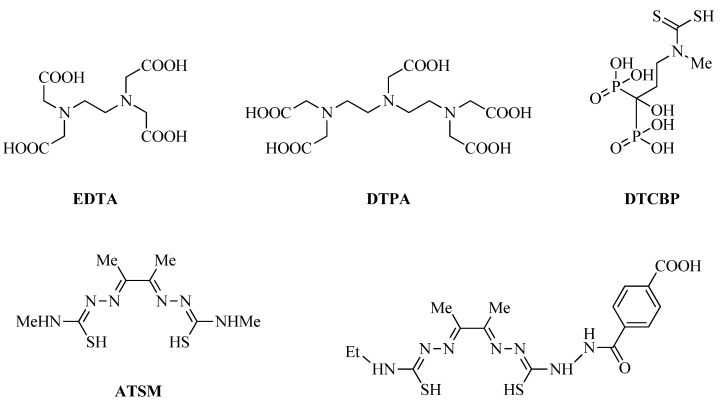
Commonly used acyclic copper chelators EDTA, DTPA, DTCBP, dithiocarbamate, and bis(thiosemicarbazone) derivatives.

As non-cyclic chelators, ethylenediaminetetraacetic acid (EDTA), diethylenetriamine pentaacetate (DTPA), dithiocarbamatebisphosphonate (DTCBP) derivatives dithiocarbamate-based ligands such as bis(thiosemicarbazone), and ATSM are also commonly used [34,37,38,39] (Figure 3).

Below, we summarize the bifunctional compounds claimed as agents for the imaging of Aβ or treatment of AD by binding to Aβ and influencing metal homeostasis published since December 2020 (Table 1).

### 2.1. BFCs Based on (2-Formyl-5-Furanyl)-3-Hydroxymethylbenzofuran

**Figure 4:**
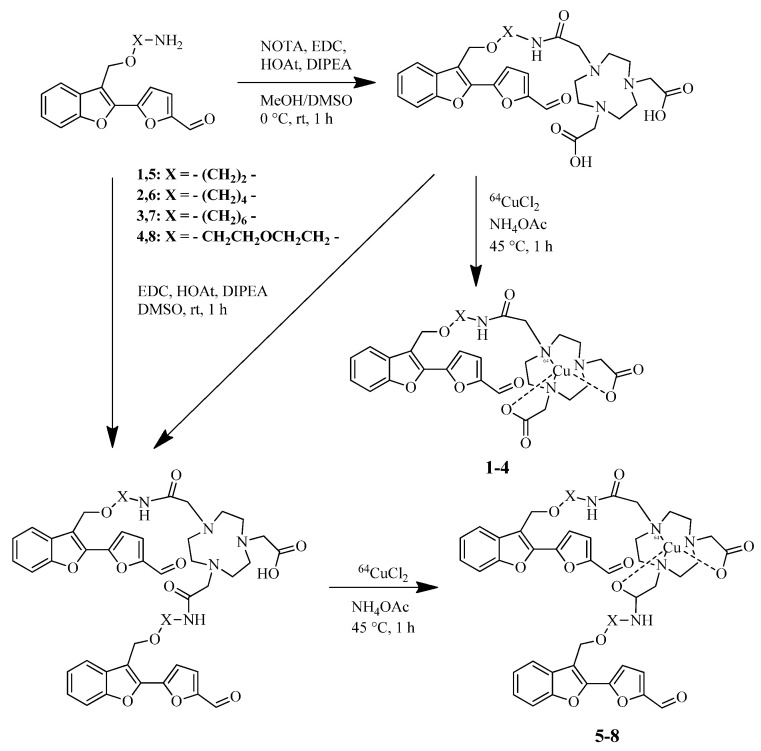
BFCs based on 2-(2-formyl-5-furanyl)-3-hydroxymethylbenzofuran scaffold with NOTA as copper chelating moiety **1–8** and Cu(II) complexes **1-Cu–8-Cu** based on them reported by Cho et al. [40].

Cho et al. reported a BFCs based on 2-(2-formyl-5-furanyl)-3-hydroxymethylbenzofuran scaffold with NOTA as copper chelating moiety [40] (Figure 4). Importantly, this molecular structure has not been used previously for developing ^64^Cu-based PET imaging agents for the Aβ aggregates relevant to AD.

To evaluate the affinity of these compounds toward amyloid plaques, a staining of nonradioactive Cu complexes **5-Cu–8-Cu** with brain sections of 3-month-old 5xFAD mice was performed and showed that complexes **5-Cu–8-Cu** bind specifically to the amyloid plaques. In addition, immunostaining with the AF594-conjugated HJ3.4 antibody (AF594-HJ3.4) revealed a good colocalization of **6-Cu** and **7-Cu** with antibody-labeled Aβ plaques.

Further, a comparison of autoradiography images of the 5xFAD mouse brain sections incubated with the divalent **6-Cu–7-Cu** and the monovalent **2-Cu–3-Cu** compounds showed that the signal intensities of the divalent compounds were higher than those of the monovalent compounds; these results support the multivalent strategy in our BFC.

Cytotoxicity of the nonradioactive Cu complexes **5-Cu–8-Cu** on neuroblastoma Neuro-2a cells was evaluated, and no cytotoxicity up to 10 μM was revealed. A brain uptake and in vivo biodistribution of the ^64^Cu complexes **5-Cu–8-Cu** in WT mice (CD-1) was also evaluated: complexes **6-Cu** and **8-Cu** exhibited high brain uptake at 2 and 60 min, with low nonspecific accumulation in the major organs. A comparison of PET/CT images of WT and 5xFAD mice injected with∼3-MBq doses of **6-Cu** and **8-Cu** showed lower intensity of signal in WT mouse brains than in the 5xFAD mouse brains, and a statistically significant higher brain uptake in the 5xFAD mice was observed for **6-Cu**.

### 2.2. Distyrylbenzene-Vanilin BFC

**Figure 5:**
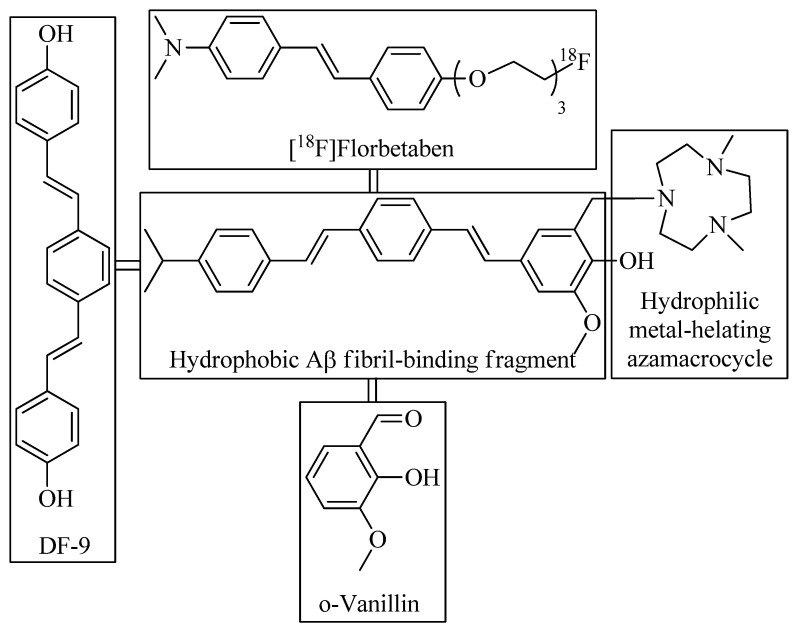
Design strategy and structure of the amphiphilic compound **9**.

**Figure 6:**
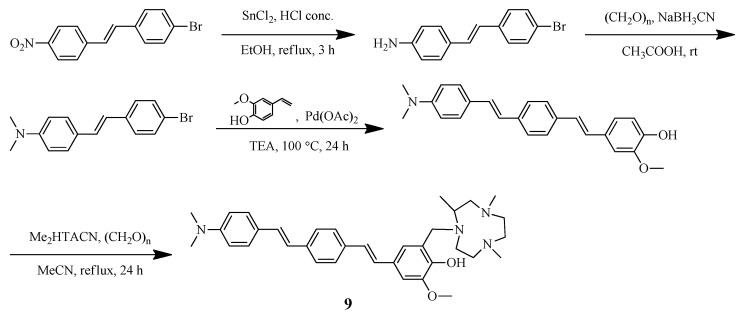
Distyrylbenzene-based bifunctional chelator **9** reported by Sun et al. [41].

Sun et al. reported [41] a distyrylbenzene-based hybrid **9** with a hydrophilic triazamacrocycle chelating moiety (Figure 5 and Figure 6). An asymmetric distyrylstilbene was designed as an FDA-approved PET imaging agent [^18^F]florbetaben. The symmetric distyrylbenzene structure of previously described compound DF-9 have been widely used in detecting amyloid plaques [49] as well as the 2-methoxy-phenol fragment reminiscent of o-vanillin that was shown to inhibit the formation of Aβ oligomers and also exhibit antioxidant properties [50].

Antioxidant ability of hybrid **9** was confirmed by trolox-equivalent antioxidant capacity (TEAC) assay. Both **9** and Cu(II) coordination compound based on **Cu-9** showed the fluorescence turn-on effect in the presence of Aβ species, especially in the presence of soluble Aβ_42_ oligomers. Importantly, in the absence of the hydrophilic azamacrocycle fragment, the binding affinity of Pre-9 toward the amyloid species dramatically decreased.

A nanomolar affinity of **9** for Aβ_42_ oligomers (Kd = 50 ± 9 nM) and Aβ fibrils (Kd = 58 ± 15 nM) was established. In addition, in the presence of both Aβ_42_ and Cu^2+^, hybrid **9** proved the ability to rescue the viability of N2a cells and significantly alleviate the neurotoxicity of Cu^2+-^ Aβ_42_ species. While monitoring of kinetics of Aβ_42_ aggregation in the presence of chelator **9** and complex **Cu-9**, an unusual behavior of ligand **9** and complex **Cu-9** was observed. Thus, hybrid **9** was found to detect the “on-pathway” Aβ_42_ oligomers, that is, monomeric Aβ_42_ aggregates, and a decrease in its fluorescence was detected when Aβ_42_ fibrils were formed in solution. This is an important result, as high-soluble Aβ oligomers have been shown to be involved in synapse loss and neuronal injury [51].

Fluorescence staining of chelator **9** with brain sections from 7-month-old 5xFAD mice was also performed, with Congo Red dye, HJ3.4. antibody, or Aβ oligomer-specific monoclonal antibody (OMAB), which specifically binds to Aβ oligomers as controls. Both **9** and **Cu-9** showed excellent colocalization with the immunofluorescence with both OMAB and HJ3.4, thus proving an ability of **9** to bind both the Aβ oligomers and fibrils in AD brain sections. In addition, a successive treatment of Aβ fibrils with Cu^2+^ and **9** lead to a significant inhibition of ascorbate consumption when compared to Aβ fibrils threated with Cu^2+^ only. Hybrid **9** found to reduce the neurotoxicity of Cu^2+^-Aβ_42_ species.

In vivo BBB permeability of **9** was also confirmed. Thus, after administration of **9** daily (1 mg/kg) to 7-month-old 5xFAD mice for 10 days via intraperitoneal injection, a strong fluorescence of mouse brain sections was detected, which was in a good colocalization with Congo Red fluorescence, and both HJ3.4 and OMAB antibodies. To assess therapeutic efficacy, 5xFAD mice were treated with **9**, a significant reduction of both amyloid plaques and associated p-tau aggregates was detected, and microglia activation was also reduced. Finally, a radiolabeled **^64^Cu-9** was synthesized, and a series of PET imaging and biodistribution studies were performed. The results obtained proved **^64^Cu-9** complex can cross the BBB and binds to the amyloid plaques. What is more important, **^64^Cu-9** proved to accumulate to a significantly larger extent in the 5xFAD mice brains vs. the WT controls.

Finally, the effect of chelator **9** on the aggregation of p-tau protein and the activation of microglia as a neuroinflammatory response was assessed using fluorescently labeled AT8 antibody, which is specific to p-tau aggregates. The total amount of p-tau aggregates surrounding the amyloid plaques was decreased in the **9**-treated vs. vehicle-treated 5xFAD mice. The level of activated microglia cells in AD mice was assessed using CF594-labeled ionized calcium-binding adapter molecule 1 (Iba1) antibody, and the ability of chelator **9** to suppress the activation of microglia cells to alleviate the neuroinflammation was revealed. Docking studies of binding of chelator **9** to both soluble Aβ oligomers and Aβ fibrils showed an ability of **9** to efficiently restrict the fibril formation in vivo, probably due to the preferential binding to the fibril ends of **9** to mitigate the Aβ elongation process.

### 2.3. Benzothiazole-Based BFCs

**Figure 7:**
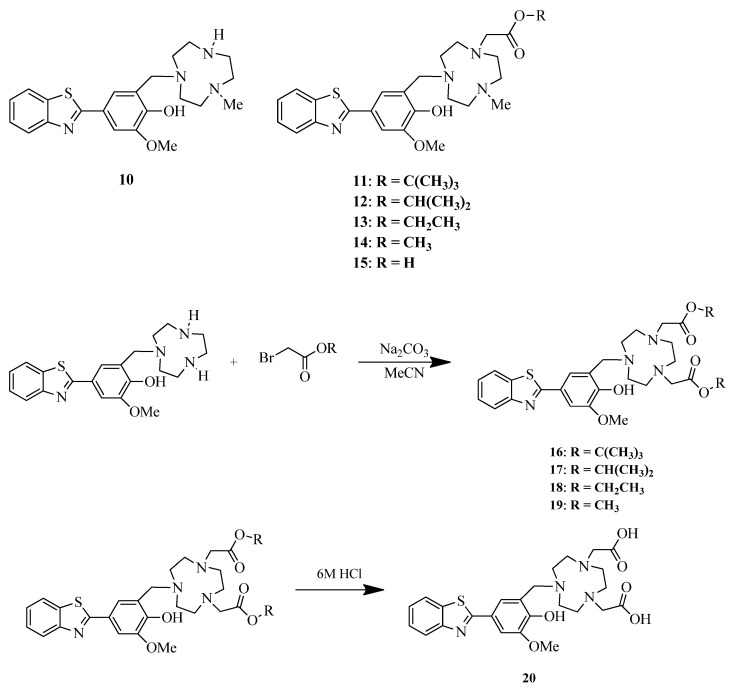
Benzothiazole-based BFCs **11–15** with ester derivatives of TACN, non-ester derivative **10** reported; BFCs **16–20** with two ester moieties of TACN reported by Wang et al. [42,43].

Wang et al. reported five benzothiazole-based BFCs **11–15** with ester derivatives of TACN and non-ester derivative **10** [42] (Figure 7). Ester derivatives of the carboxylate pendant arm were conjugated with TACN moiety in order to increase the lipophilicity of the bifunctional chelators and facilitate brain uptake. Spectrophotometric titrations were used to quantify a stability constant of the complexes (log Ks); the results show that a carboxylic acid or ester moieties in TACN scaffold increases the log K by 3−4 orders of magnitude versus the parent TACN derivative.

Fluorescence imaging of amyloid plaques in 5xFAD mouse brain sections as well as immunostaining with HJ3.4 antibody revealed a specific binding of BFCs **11, 13, 14,** and their Cu(II) complexes to Aβ species. A specific binding of ligands and their Cu(II) complexes with amyloid plaques was confirmed by staining with Congo Red dye on brain sections collected from 11-month-old 5xFAD mice. In addition, good colocalization of both ligands and their Cu(II) complexes was shown on brain sections from six-month-old 5xFAD mouse with HJ3.4 antibody (AF594- HJ3.4), especially for BFCs **11, 13, 14**.

Autoradiography studies were performed on brain sections from 11-month-old 5xFAD and aged-matched WT mice. The results obtained strongly suggest that the ^64^Cu-labeled BFCs exhibited the ability to detect Aβ species ex vivo, and TACN esters show more specific binding to Aβ plaques than corresponding acids. In vivo biodistribution experiments in CD-1 mice were also performed to investigate the pharmacokinetics and revealed some brain uptake of complexes. The highest brain uptake was shown by **^64^Cu-14** of 0.46 ± 0.21% ID/g at 2 min post-injection.

In addition, the same scientific group reported five benzothiazole-based complexes with TACN chelator with two ester moieties [43] (Figure 7). A direct binding of **20** with Aβ_42_ fibrils was confirmed by fibril titration with solution of **20**, a saturation behavior was observed, and a binding constant was calculated (Kd = 121 ± 44 nM).

A co-staining with a brain sections of 11-month-old 5xFAD transgenic mice with Congo Red dye revealed affinity of BFCs **17** and **18** and their Cu(II) complexes **17-Cu** and **18-Cu** toward Aβ species, and the specific staining of **Cu-20** with AF594-conjugated HJ3.4 antibody (AF594-HJ3.4) also exhibited a strong colocalization with the antibody stained regions. Autoradiography studies of 11-month-old 5xFAD and age-matched WT mice revealed an increased intensity that **^64^Cu-20** exhibits in 5xFAD mice compared to WT.

**Figure 8:**
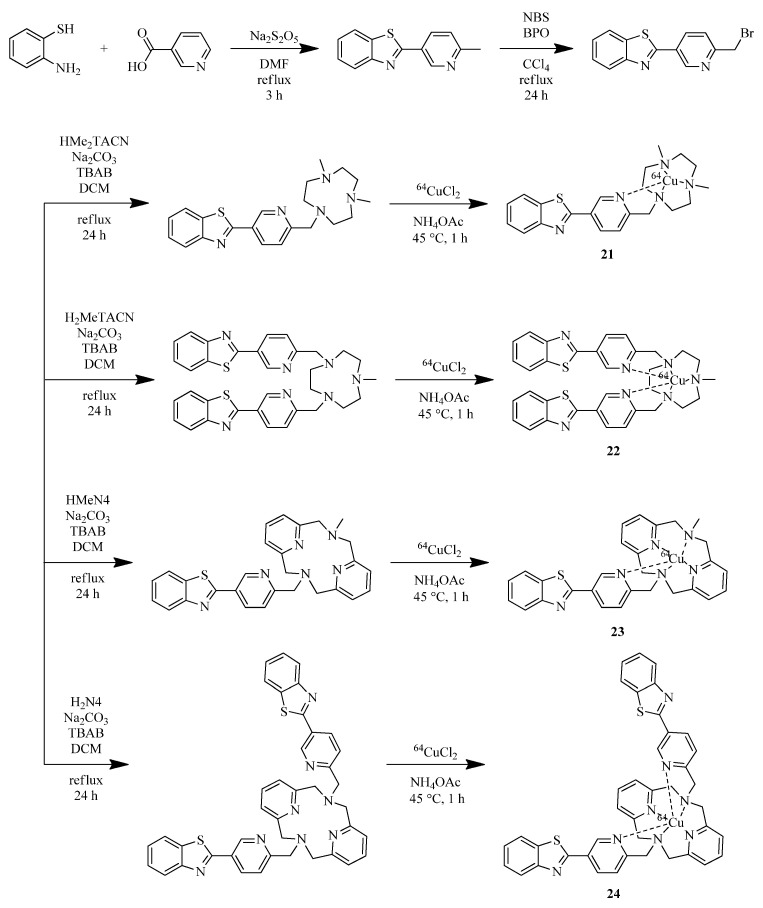
Benzothiazole-based BFCs **21–24** and Cu(II) complexes based on them, **21-Cu–24-Cu,** reported by Huang et al. [44].

Huang et al. reported Benzothiazole-based complexes with copper-chelating TACN and 2,11-diaza [3.3]-(2,6)pyridinophane (N4) moieties **21–24** [44] (Figure 8).

EPR spectra of complex **Cu-22** suggest that the complex remains mononuclear in solution. Fluorescence imaging studies on 5xFAD mouse brain sections treated with **21–24**, and Cu(I,II) complexes based on them revealed a specific binding of ones to Aβ plaques, which was confirmed by co-staining CF594-conjugated HJ3.4 antibody (CF594-HJ3.4), affinic to a wide range of Aβ species. Autoradiography studies of ^64^Cu-labeled **21–24** complexes revealed a specific binding of the complexes to amyloid plaques, which was also confirmed by blocking with the nonradioactive blocking agent B1. A great contrast between the intensity of WT and 5xFAD mice brains for all radiolabeled complexes was shown, especially for **^64^Cu-22.** In addition, an incubation of **^64^Cu-22** and**^64^Cu-23** with human serum at 37 °C for up to 24 h showed the stability of the complexes. To evaluate the ability of radiolabeled coordination compounds to cross the BBB in vivo, a biodistribution in normal CD-1 mice was evaluated. The highest brain uptake for **^64^Cu-22** complex was shown and was approximately ~ 0.4% both after 2 min post injection and after 24 h, which indicates the rapid penetration of the complex into the brain and its retention.

**Figure 9:**
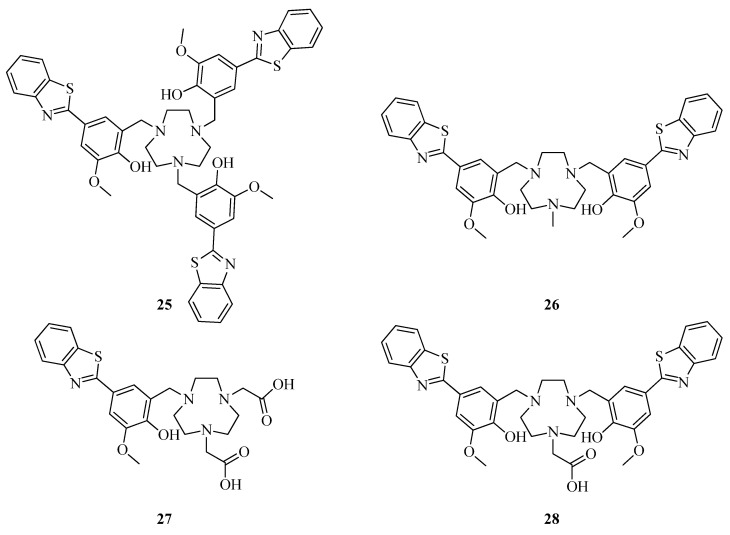
Benzothiazole-based BFCs **25–28** reported by Wang et al. [45].

Wang et al. investigated a series of BFCs with an Aβ-binding 2-(4-hydroxyphenyl)-benzothiazole moiety and metal-chelating 1,4,7-triazacyclononane (TACN) ligands and gallium coordination compounds based on them [45] (Figure 9).

Histological staining of 5xFAD mouse brain sections with compounds **25–28** showed a good affinity of BFCs **25, 26, 28** for the amyloid aggregates, which correlated well with Congo Red or HJ3.4 antibody controls. In contrast, BFCs **27** exhibited weak Congo Red colocalization, thus indicating that introduction of extra amyloid β targeting moieties are able to increase the affinity of BFCs to amyloid plaques. Autoradiography studies with radiolabeled complexes **[^68^Ga]25–28** revealed a specific binding with brain sections of 5xFAD and WT mice, with the highest non-specific binding of ^68^Ga-labeled bivalent complexes.

**Figure 10:**
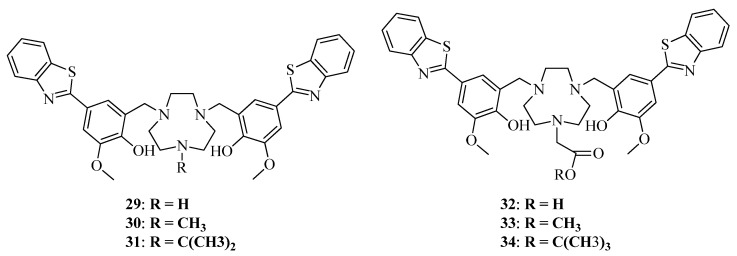
Benzothiazole-based BFCs **29–34** reported by Terpstra et al. [46].

Recently, the same scientific group reported a series of BFCs containing two Aβ-targeting fragments and a TACN macrocyclic ligand and novel derivatives with carboxylate ester arms (Figure 10) [46]. ThT competition assays revealed binding of BFCs to Aβ plaques with most active hybrids **30, 31**. In ex vivo autoradiography studies of ^64^Cu-radiolabeled BFCs with brain sections from 11-month-old 5xFAD and aged-matched WT mice, BFCs **29, 32, 33** exhibited ∼4-fold increase for 5xFAD vs. WT brain sections, with hybrid 36 exhibiting the highest overall intensity. Finally, **^64^Cu-30** showed the most promising brain uptake in CD-1 mice, with a maximum %ID/g of 0.47 ± 0.12 at 2 min post-injection.

### 2.4. Azo-Stilbene-Based BFCs

**Figure 11:**
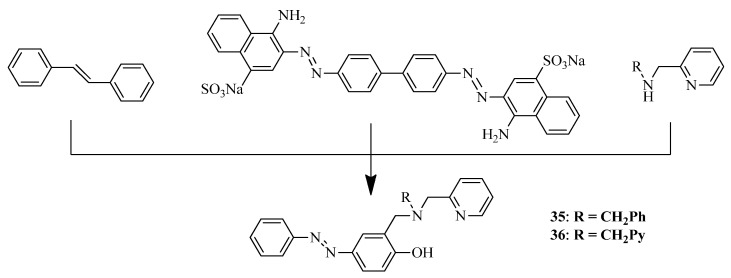
Azo-stilbene-based BFCs **35, 36** with two ester moieties of TACN reported by Rana et al. [47].

Rana et al. reported unusual bifunctional compounds that include the amyloid binding properties from stilbene and the staining characteristics of Congo Red, a commonly used Aβ-specific dye, conjugated with strong metal-binding arms [47]. These BFCs were designed to target metal-mediated neurotoxicity, but may also be considered as a perspective of organic scaffolds for design of metal-based drugs for PET Aβ imaging. Azo-stilbene-derived compounds with N,N,O and N,N,N,O donor metal chelation moiety were designed and thoroughly investigated (Figure 11).

An ability of BFCs **35, 36** to bind Aβ plaques was confirmed using ThT competition assay as well as UV−Vis spectroscopy. Inhibition of Aβ_42_ aggregation by BFCs **35, 36**, as well as **Cu-35** and **Cu-36** was monitored by a decrease in ThT fluorescence. Aβ_42_ monomers showed low ThT fluorescence and a striking increase in fluorescence during aggregation. Both compounds **35** and **36** reduced the fluorescence of Aβ_42_ aggregates as well as Aβ_42_ aggregates pretreated with Cu^2+^ or Zn^2+^. Inhibition of Aβ_42_ metal-free and metal−Aβ aggregation was also confirmed by TEM images. Thus, in the presence of BFCs **35, 36**, the morphology was quite different from that with Aβ_42_ alone. In addition, Aβ_42_ aggregation in the presence of both Cu^2+^ or Zn^2+^ and chelators **35** and **36** led to lesser aggregates of amorphous morphology, differing from that of Aβ_42_ alone.

Docking interactions of **35** and **36** with the Aβ_40_ fibrillar structure revealed their positioning near the KLVFF hydrophobic region of the peptide, π−π interactions of BFCs **35, 36** with both with Aβ_40_ and Aβ_42_. In addition, molecular docking with acetylcholinesterase AChE showed an interaction of BFCs **35, 36** with catalytic active site (CAS) and peripheral anionic site (PAS) of AChE. An ability of cholinesterase inhibition was also confirmed (Table 2), as well as the ability of BFCs **35, 36** to inhibit AChE-induced Aβ_42_ aggregation confirmed by ThT fluorescence assay.

An antioxidant property of BFCs **35, 36** was confirmed using 6-hydroxy-2,5,7,8-tetramethylchroman-2-carboxylic acid (Trolox) (TEAC). Finally, compound **35** showed low neurotoxicity on Neuro2A cells, in contrast to **36**, thus suggest that extra pyridine groups may lead to higher cell toxicity.

### 2.5. Styrylpyridyl-Based BFCs

**Figure 12:**
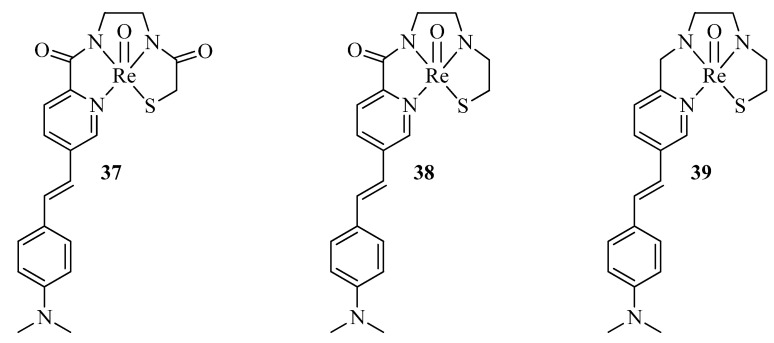
Styrylpyridyl-based BFCs **37–39,** reported by Spyrou et al. [48].

Spyrou et al. reported a three tetradentate ligand based on styrylpyridyl scaffolds with pyridyl, amide, amine, and thiol chelating moieties and charge-neutral complexes [Tc=O]^3+^ and [Re=O]^3+^ based on it [48] (Figure 12).

The ability of BFCs **37–39** to interact with Aβ_1−40_ fibrils was investigated with a competition assay between each rhenium complex and ThT. Each of the complexes showed an ability to displace ThT from the fibrils and had significant affinity for Aβ_1−40_ with Ki ∼ 240−260 nM. In addition, excellent colocalization of the complexes with Aβ plaques of human brain tissue was revealed by immunohistochemistry with a Aβ-specific 1E8 antibody as a control. Radiolabeled [^99m^Tc][TcO **37–39**] were obtained, and biodistribution of [^99m^Tc][TcO **37–39**] in wild-type mice was determined. Unfortunately, brain uptake values of the complexes were too low for SPECT imaging.

## 3. Conclusions

Summarizing the above data, one can conclude that metal-containing imagining agents are a promising alternative to clinically used radiopharmaceuticals based on short-lived ^11^C and ^18^F isotopes. This review provides examples of the successful design of ligands and coordinating compounds based on them, capable of crossing the blood-brain barrier and successfully binding to amyloid plaques in an AD brain. Radiolabeled complex **6-^64^Cu** showed a significant higher brain uptake in the 5xFAD mice than in WT; this testifies to the thoughtful drug design and confirms the ability of a Cu-based coordination compounds to act as imaging agents for Aβ plaques.

In addition, the successful design of an effective and selective bifunctional chelator **9** and a coordination compound **Cu-9** based on it shows great potential of copper-containing coordination compounds as drugs for imaging of Alzheimer’s disease. In addition, hybrid **9** showed the ability to act on soluble Aβ oligomers, which is an extremely promising result due to the high toxicity of the latter, as well as an acute shortage of drugs capable of acting on them. During several attempts to create coordination compounds with an ester or carboxyl group, radiolabeled coordination compound **^64^Cu-20** showed increased brain uptake in 5xFAD mice compared to WT. It should also be noted that the ability of bifunctional ligands **35, 36** to inhibit acetylcholinesterase suggests that the developed bifunctional compounds can be not only effective imaging agents, but also have therapeutic anti-AD efficacy.

Thus, bifunctional compounds with an amyloid affinity fragment together with a chelating fragment are able to visualize both Aβ plaques and soluble Aβ oligomers. Their ability to influence metal homeostasis and Aβ aggregation opens up opportunities for creating not only visualizing but also theranostic agents for AD.

## Figures and Tables

**Table 1:** Multifunctional chelators for visualization of Aβ plaques.

BFCs	Metal	Imaging Method	Amyloid-Binding Moiety	Chelator	Brain Uptake, ID/g **, Time Post Injection	Ref.
**1–8**	Cu	PET *	Benzofuran	NOTA	2-, 60-, and 240-min p.i. *** 1 0.65 ± 0.23 0.10 ± 0.03 0.05 ± 0.00 2 0.76 ± 0.03 0.35 ± 0.10 0.08 ± 0.00 3 0.38 ± 0.04 0.13 ± 0.02 0.08 ± 0.01 4 0.83 ± 0.14 0.27 ± 0.05 0.09 ± 0.02	[40]
**9**	Cu	PET	Florbetaben + Vanilin	TACN	WT: 0.75 ± 0.10% ID/g 2 min 18 ± 0.02% ID/g 1 h AD mice: 0.79 ± 0.06%ID/g 2 min 0.39 ± 0.02% ID/g (1 h)	[41]
**10–15**	Cu	PET	Benzothiazole	TACN with one alkyl carboxylate ester pendant arms	2 min, 1 h, 4 h 11 0.35 ± 0.01 0.04 ± 0.01 0.03 ± 0.01 12 0.23 ± 0.06 0.02 ± 0.01 0.01 ± 0.00 13 0.32 ± 0.02 0.02 ± 0.00 0.01 ± 0.00 14 0.46 ± 0.21 0.14 ± 0.00 0.18 ± 0.02 15 0.23 ± 0.05 0.02 ± 0.02 0.02 ± 0.00	[42]
**16–20**	Cu	PET	Benzothiazole	TACN with two alkyl carboxylate ester pendant arms	-	[43]
**21–24**	Cu	PET	Benzothiazole	1,4,7-triazacyclononane (TACN) and 2,11-diaza [3.3]-(2,6)pyridinophane (N4)	Cu-23: 0.2% ID/g at 2 min, yet an increased brain accumulation of ∼0.4% ID/g was observed after 4 h	[44]
**25–28**	Ga	PET	2-(4-hydroxyphenyl)-benzothiazole	TACN	0.10 ± 0.03 0.05 ± 0.02 (2 h) 0.26 ± 0.12 0.07 ± 0.02 0.03 ± 0.00 0.33 ± 0.12 0.01 ±0.009 (2 h)	[45]
**29–34**	Cu	PET	Benzothiazole	TACN	0.47 ± 0.12 (2 min)	[46]
**35, 36**	-	-	Azo-stilbene	Pyridine	-	[47]
**37–39**	Tc	SPECT ****	Styrylpyridyl	Diamide−thiol, Monoamide−monoamine− thiol Diamine−thiol	WT: ***** [^99m^Tc][TcO-38] 2 min 0.15 ± 0.06% 35 min 0.17 ± 0.01% [^99m^Tc][TcO-39] 2 min 0.36 ± 0.09% 35 min 0.15 ± 0.02%	[48]

* PET—positron emission tomography, ** ID/g—injected dose per gram of tissue, p.i. ***—post-injection, **** SPECT—single-photon emission computerized tomography, ***** WT—wild-type mice.

**Table 2:** In vitro AChE inhibition by BfCs **35, 36**.

AChE	35	36	Rivastigmine	Dopenezil
IC_50_ (µM)	4.18 ± 0.15	3.86 ± 0.13	11.02 ± 1.26	0.06 ± 1.13

## Abbreviations

AD—Alzheimer’s disease, AChE—acetylcholinesterase, TEAC—trolox-equivalent antioxidant capacity, WT—wild type mice, SPECT—single-photon emission computerized tomography, BFCs—bifunctional compounds, CR—Congo Red, CAS—catalytic active site, PAS—peripheral anionic site, PET—positron emission tomography, MRI—magnetic resonance imaging, TACN—2,4,7-triazacyclononane, NOTA—1,4,7-triazacyclononane-1,4,7-triacetic acid, TETA—1,4,8,11-tetraazacyclotetradecane-N,N′,N′′,N′′′-tetraacetic acid, DOTA—2,2′,2′′,2′′′-(1,4,7,10-tetraazacyclododecane-1,4,7,10-tetrayl)tetraacetic acid
